# Mint Oils: In Vitro Ability to Perform Anti-Inflammatory, Antioxidant, and Antimicrobial Activities and to Enhance Intestinal Barrier Integrity

**DOI:** 10.3390/antiox10071004

**Published:** 2021-06-23

**Authors:** Monika Hejna, Lauren Kovanda, Luciana Rossi, Yanhong Liu

**Affiliations:** 1 Department of Health, Animal Science and Food Safety, Università degli Studi di Milano, Via Trentacoste 2, 20134 Milan, Italy; monika.hejna@unimi.it; 2 Department of Animal Science, University of California, Davis, 4302 Meyer Hall, One Shields Ave, Davis, CA 95616, USA; llkovanda@ucdavis.edu

**Keywords:** anti-inflammatory, antioxidant, antimicrobial, mint oils, pigs, transepithelial electrical resistance

## Abstract

The objectives of the study were to test the biological activities of peppermint and spearmint oils via (i) measuring in vitro anti-inflammatory effects with porcine alveolar macrophages (PAMs), (ii) determining the barrier integrity of IPEC-J2 by analyzing transepithelial electrical resistance (TEER), (iii) testing their antioxidant activities, and (iv) investigating the antimicrobial activity against enterotoxigenic *Escherichia coli* (ETEC) F18+. Briefly, (i) macrophages were seeded at 10^6^ cells/mL and treated (24 h) with mint oils and lipopolysaccharide (LPS). The treatments were 2 (0 or 1 μg/mL of LPS) × 5 (0, 25, 50, 100, 200 µg/mL of mint oils). The supernatants were collected for TNF-α and IL-1β measurement by ELISA; (ii) IPEC-J2 cells were seeded at 5 × 10^5^ cells/mL and treated with mint oils (0, 25, 50, 100, and 200 μg/mL). TEER (Ωcm^2^) was measured at 0, 24, 48, and 72 h; (iii) the antioxidant activity was assessed (0, 1, 50, 100, 200, 500, and 600 mg/mL) using the 2,2-diphenyl-1-picrylhydrazyl (DPPH) radical scavenging and reducing power assays; (iv) overnight-grown ETEC F18+ were quantified (CFU/mL) after supplementing with peppermint and spearmint oils (0, 1.44, 2.87, 5.75, 11.50, and 23.00 mg/mL). All data were analyzed using the MIXED procedure. Both mint oils significantly inhibited (*p* < 0.05) IL-1β and TNF-α secretion from LPS-stimulated PAMs. Mint oil treatments did not affect TEER in IPEC-J2. Spearmint and peppermint oils exhibited (*p* < 0.05) strong antioxidant activities in DPPH and reducing power assays. Both mint oils also dose-dependently inhibited (*p* < 0.05) the growth of ETEC F18+ in vitro. The results of the study indicated that both mint oils are great candidate feed additives due to their in vitro anti-inflammatory, antioxidant, and antimicrobial effects. Further research is needed to evaluate their efficacy in vivo.

## 1. Introduction

Post-weaning is a critical phase in swine production, where piglets are exposed to a combination of stressors [1] that are associated with fluctuations in gut function and cause different multifactorial diseases [2]. Due to the increase in antibiotic resistance [3], antibiotics as growth-promoting agents have been banned in the EU since 2006 [4]. The first adopted alternative to in-feed antibiotics was the widespread application of high doses of zinc oxide, which, despite their antibacterial and anti-inflammatory activities, raised concerns related to environmental pollution [5,6]. The use of high-dose zinc in feed may also have contributed to the emergence of methicillin-resistant *Staphylococcus aureus* (MRSA) as this metal is associated with the co-selection of resistance genes to antibiotics [7,8]. Thus, the EU also recently banned the inclusion of pharmacological levels of zinc oxide in animal feed after 2022 [9]. The role of nutrition and novel functional feed additives and ingredients requires urgent consideration in terms of reducing the use of antibiotics, in order to improve the profitability and to increase the sustainability of agriculture.

Plant extracts are secondary plant metabolites and can be obtained naturally from parts of plant materials, such as flowers, buds, seeds, leaves, twigs, bark, wood, fruits, and roots. Plant extracts are composed of two different forms: liquid oil and solid powder. Liquid plant extracts are water-insoluble and are often referred to as essential oils [10]. Plant extracts are of potential interest because of their anti-inflammatory, antioxidant, and antimicrobial activities [11,12,13,14,15,16]. Peppermint (PM, *Mentha piperita* L.) and spearmint (SM, *Mentha spicata* L.) belong to the mint (*Mentha*) genus and are known for their high content of essential oils deposited in the glandular trichomes [17,18] and the abundance of phenolic compounds [14,19]. The activities of mint oils are associated with their individual chemical compositions and different phytochemical constituents that influence their biological functions [10]. Mint oils mostly contain phenolic compounds and flavonoids [20,21,22,23,24] and their therapeutical activities can be thus considered in the treatment of various diseases, such as digestive disorders, diarrhea, intestinal inflammation, and nervous system disorders, in humans and animals [17,20]. Mint oils and their therapeutic potential as feed additives are therefore under investigation for the promotion of health in piglets due to their anti-inflammatory, antioxidant, and antimicrobial properties [25].

In the present study, several in vitro models were adopted to thoroughly evaluate the biological activities of peppermint and spearmint oils. Porcine alveolar macrophages (PAMs) are lung tissue-resident professional phagocytes that play important roles in the immune responses of pigs [26,27]. Lipopolysaccharide (LPS) challenge could induce the secretion of pro-inflammatory cytokines from PAMs [13,28]. Thus, culturing PAMs with LPS challenge has been widely used to test the in vitro anti-inflammatory effects of different bioactive compounds [29,30]. The IPEC-J2 cell line is generated from intestinal porcine enterocytes that are isolated from the middle of the jejunum of neonatal piglets [31] and is commonly used to conduct in vitro research focusing on the intestinal physiology of pigs. When inflammation is enduring, IPEC-J2 cells must form a polarized monolayer in order to maintain barrier function [32]. Measurement of transepithelial electrical resistance (TEER) across IPEC-J2 monolayers is a valuable tool to evaluate the intestinal barrier integrity in vitro [33]. Furthermore, several chemical-based assays, including the 2,2-diphenyl-1-picrylhydrazyl (DPPH) radical scavenging and reducing power assays, have been widely used to assess antioxidant activities.

The antimicrobial activity of essential oils, particularly mint oils, has been widely evaluated with many pathogenic bacteria due to their bioactive compounds [34,35,36,37,38]. The current study targeted Enterotoxigenic *Escherichia coli* (ETEC) F18, which is one of the most important pathotypes causing post-weaning diarrhea and thus increasing the use of antibiotic treatment in weaned pigs [39,40].

Therefore, the aims of the current study were to test the in vitro biological activities of peppermint and spearmint oils via (i) measuring in vitro anti-inflammatory effects with PAMs, (ii) measuring the barrier integrity of IPEC-J2 by analyzing TEER, (iii) testing the antioxidant activities with two chemical-based assays, and (iv) investigating the antimicrobial activity against ETEC F18+.

## 2. Materials and Methods

### 2.1. Materials

Peppermint oil (catalog ID: PP0060) was purchased from Ward’s Science (Rochester, NY, USA) and spearmint oil (catalog ID: SP306) was purchased from Spectrum Chemicals & Corp (Gardena, CA, USA). Both oils were 100% pure and no extraction method was used before further experiments. To prepare cell culture treatment, mint oils were first dissolved in dimethyl sulfoxide (DMSO) and were further diluted with sterile DMEM/F12 (Dulbecco’s Modified Eagle Medium) or RPMI-1640 (Roswell Park Memorial Institute) medium for culturing IPEC-J2 or porcine alveolar macrophages, respectively. Culture media contained 1% antibiotics (100 mg/mL penicillin and 100 mg/mL streptomycin; Mediatech, Inc., Manassas, VA, USA) and 5% fetal bovine serum (HyClone Laboratories, Inc., Logan, UT, USA). The final concentration of DMSO in media did not exceed 0.05%. In the antioxidant assays, mint oils were prepared by diluting the stock solution with methanol (*w/v*). In the antimicrobial assays, mint oils were dissolved in DMSO and then further diluted in Luria Bertani broth (LB).

### 2.2. In Vitro Anti-Inflammatory Assays

#### 2.2.1. Collection of Porcine Alveolar Macrophages

The protocol for PAM collection was approved by the Institutional Animal Care and Use Committee (IACUC #20809) at the University of California, Davis. Porcine alveolar macrophages were collected from six healthy pigs at 7 weeks of age and around 12 kg of body weight (BW). Pigs were anesthetized by intramuscular injection of a 1 mL combination of telazol, ketamine, and xylazine (2:1:1) per 23.3 kg of BW. The final mixture contained 100 mg telazol, 50 mg ketamine, and 50 mg xylazine in 1 mL (Fort Dodge Animal Health, Fort Dodge, IA, USA). After anesthesia, pigs were euthanized by intracardiac injection with 78 mg sodium pentobarbital (Sleepaway; Henry Schein, Inc., Indianapolis, IN, USA) per 1 kg of BW. Porcine alveolar macrophages were collected from bronchoalveolar lavage following the procedures described by Baarsch et al. [41]. Briefly, lungs with intact trachea were removed immediately after euthanizing pigs and 100 mL of ice-cold phosphate-buffered saline (PBS) without calcium or magnesium was poured into them through the trachea. After massaging the lungs for around 60 s, the lavage fluid was filtered through a double layer of sterile gauze into 50 mL conical centrifuge tubes and then pelleted by centrifuging at 400× *g* for 10 min at room temperature. The pelleted cells were washed with 4 mL ACK lysing buffer (Thermo Fisher, Waltham, MA, USA) to lyse red blood cells and were suspended in 5 mL of RPMI-1640 culture medium. Live cells were stained by trypan blue dye exclusion (Sigma-Aldrich Co., St. Louis, MO, USA) and were counted using a hemocytometer (Fisher Scientific, Inc., Pittsburgh, PA, USA). The final cell concentration was adjusted to 1 × 10^6^ cells/mL. The viability of cells was greater than 97%. In the present study, the term “porcine alveolar macrophages” is used because the majority of bronchoalveolar lavage fluid cells are macrophages [42].

#### 2.2.2. Cell Culture and Experimental Design

Porcine alveolar macrophages were cultured in 24-well or 96-well cluster plates at a density of 10^6^ cells/mL. All plates were incubated overnight at 37 °C in a humidified 5% CO_2_ incubator to allow macrophages to adhere to the bottom. The nonadherent cells were washed away with pre-warmed RPMI-1640 medium. Adhered macrophages were treated in duplicate with fresh pre-warmed RPMI-1640 medium containing different treatments. After another 24 h incubation, supernatants in duplicate were collected, pooled, and stored at −80 °C for cytokine analysis. Peppermint oil and spearmint oil were tested with the same experimental design as a 2 (without or with 1 μg of LPS/mL) × 5 (5 different doses of mint oil) factorial arrangement in a randomized complete block design. Therefore, there were a total of 10 treatments for each mint oil. The negative control was the treatment without either mint oil or LPS, and the positive control was the treatment without mint oil but with LPS. The doses of peppermint oil and spearmint oil tested in this experiment were 0, 25, 50, 100, and 200 μg/mL. The LPS was derived from *Escherichia coli* 0111:B4 (Sigma Co., St. Louis, MO, USA).

#### 2.2.3. Detection of Cell Viability

To determine the cytotoxicity of mint oils on porcine alveolar macrophages, the 3-(4,5-dimethylthiazol-2-yl)-2,5 diphenyltetrazolium bromide (MTT Cell Proliferation Assay Kit) assay was used (Invitrogen, Vybrant MTT Molecular Probes Inc., Eugene, OR, USA). This assay measured the metabolic activity of cells with a color reaction catalyzed by mitochondrial enzymes to detect the number of live cells [43]. Briefly, cells in 96-well plates were treated with different treatments and were cultured for 24 h as described above. After this, supernatant was removed and 100 μL of fresh RPMI-1640 culture medium and 10 μL of 12 mM MTT solution were added to each well. After 4 h of incubation, 50 μL of DMSO solution was added and thoroughly mixed. The optical density (OD) was measured at 540 nm (Synergy HTX Multi-Mode Microplate Reader, BioTek, Winooski, VT, USA) after 10 min incubation. The background signal inherent to the plates when cells were not present was subtracted from the absorbance obtained from each sample. The average OD of the negative control was calculated and set to 100%. The relative viability of each well was calculated using the following formula: (OD of treated cells/mean OD of negative control) × 100. The percentage of live cells represents both viability and proliferation.

#### 2.2.4. Measurements of Pro-Inflammatory Cytokines

Protein concentrations of TNF-α and IL-1β in cell culture supernatants were measured by commercial ELISA kits (R&D Systems, Inc., Minneapolis, MN, USA) according to the manufacturer’s instructions. Briefly, standard, control, and samples were added to the wells with coated monoclonal antibody specific for each cytokine. After incubation for 2 h, the unbound substances were washed away, and an enzyme-linked polyclonal antibody specific for the cytokine was added to the wells. A further 2 h of incubation was followed by a wash to remove any unbound antibody-enzyme reagent, and then a substrate solution was added to the wells. Color was developed in proportion to the amount of the cytokine bound in the initial step. The color development was stopped by adding stop solution (50 µL/well of stop solution with hydrochloric acid), and the intensity of color was measured at 540 nm. Concentrations of individual cytokines were calculated based on the standard curves. All samples were analyzed in duplicate.

### 2.3. Transepithelial Electrical Resistance Assay

The culture and maintenance of IPEC-J2 followed the procedures described in previous research [44]. Briefly, 0.5 mL of IPEC-J2 cells (5 × 10^5^ cells/mL) was seeded in 12-well plates with 1.12 cm^2^ polycarbonate transwell tissue culture-treated inserts (0.4 µm pores; Corning Incorporated, Lowell, MA, USA). Cells were then cultured using DMEM/F12 supplemented with 5% fetal bovine serum and 1% antibiotics and were incubated at 37 °C in a 5% CO_2_ incubator for 4 to 5 days until confluence was reached and epithelial cells differentiated (1000 Ωcm^2^). Cells were treated with different doses of mint oils (0, 25, 50, 100, and 200 μg/mL). TEER was measured (Ωcm^2^) at 0 h (before treatment) and at 24, 48, and 60 h post-treatment using a Millicell ERS-2 voltohmmeter (MilliporeSigma, St. Louis, MO, USA). The experimental design was 4 (4 time points) × 5 (5 doses of each mint oil) factorial arrangement in a randomized complete block design with 10 replicates in duplicate wells. Blank wells containing transwell inserts and culture medium were also measured at each time point. The resistance of monolayer was calculated according to the formula: R_monolayer_ [Ω] = R_sample_ − R_blank_. Resistance was inversely proportional to the area of the membrane: R_monolaye_*r* [Ω] × monolayer area [cm^2^] = R_reported_ [Ωcm^2^]. The MTT assays were also performed to determine the cytotoxicity of mint oils on IPEC-J2 using similar procedures to those described above.

### 2.4. Antioxidant Assays

DPPH radical scavenging capacity and reducing power assays were performed to evaluate the antioxidant activity of mint oils. Peppermint and spearmint oils were tested at 0, 1, 50, 100, 200, 500, and 600 mg/mL. All assays were repeated six times.

#### 2.4.1. DPPH Radical Scavenging Capacity Assay

The scavenging capacity of mint oils against 2,2-diphenyl-1-picrylhydrazyl (DPPH, Sigma, St. Louis, MO, USA) radical was determined based on the method described by Zhou et al. [45] and Wu et al. [14]. Briefly, samples were mixed with DPPH solution (25 g/mL in methanol) at a ratio of 1:39 (*v/v*). The scavenging capacity of each mint oil was calculated based on the equation: DPPH + scavenging capacity (%) = [(A_blank_ − A_test_)/A_blank_] × 100, where A_blank_ was the absorbance of the blank sample, and A_test_ was the absorbance of the test sample. Half maximal effective concentrations (EC_50_, mg/mL) of each mint oil were calculated accordingly. A lower EC_50_ indicated a higher radical scavenging capacity.

#### 2.4.2. Reducing Power Assay

The ferric iron reducing capacity of mint oils was determined following the procedures of Chung et al. [46] and Bhalodia et al. [47], with minor modifications according to Wu et al. [14]. Briefly, equal volumes of test sample, 2 M phosphate-buffered saline solution (PBS, pH 6.6), and 1% potassium ferricyanide (Sigma, St. Louis, MO, USA) were mixed thoroughly. Ascorbic acid prepared at different concentrations (0, 1, 5, 10, 50, and 100 µM) was used as the standard samples. The ferric reducing capacity was calculated as ascorbic acid equivalent, and the EC_50_ was calculated for each mint oil.

### 2.5. Escherichia coli Growth Inhibitory Activity

A liquid culture-based *Escherichia coli* O138 F18+ growth inhibition assay was performed to evaluate the inhibitory activity of peppermint oil and spearmint oil at different concentrations. One ETEC strain, harboring F18 (F18+) adhesive fimbriae, was obtained from a permanent collection of the University of Milan and previously characterized [48,49]. The bacteria were grown at 37 °C with shaking (150 × rpm) in lysogeny broth (LB) broth for 12 h prior to being used as inoculants for all experiments. Overnight-grown ETEC F18+ were inoculated in tubes containing 15 mL of LB medium supplemented with different doses (0, 1.44, 2.87, 5.75, 11.50, and 23.0 mg/mL) of spearmint and peppermint oils, respectively. Prior to inoculation, bacterial cultures were adjusted to identical density by spectrophotometry (600 nm). All tubes were incubated aerobically with shaking (150 × rpm) at 37 °C. The bacterial growth was determined via measurement of the optical density of each culture at 600 nm (OD_600_) at 60 min intervals in a spectrophotometer (680 Microplate Reader, BioRAD, Hercules, CA, USA). Bacteria-free tubes with equivalent concentrations of mint oils were used as blanks to subtract the background turbidity caused by mint–protein interactions [50,51]. All data obtained from the optical density evaluation were converted to log-transformed based cell count (CFU/mL) by a calibration curve (considering 1 OD = 10^9^ cells/mL). The curve was obtained by monitoring ETEC F18+ growth over time, in the same experimental conditions, using the classic plate counting method [52]. The assay was performed in three biological replicates and eight technical replicates.

### 2.6. Statistical Analysis

All data generated from different assays were analyzed by ANOVA using the MIXED procedure (SAS 9.4, SAS Institute Inc., Cary, NC, USA) with different statistical models. The statistical model of the PAM assays included LPS challenge, doses of mint oils, and their interaction as fixed effects and block as random effect. In the TEER assays, a pool of 10 wells was considered an experimental unit. The statistical model included the effects of dose as fixed effect and plate as random effect. *Escherichia coli* growth data (OD_600_) were log_10_ transformed prior to statistical analysis. The model included treatments, time, and time × treatment as fixed effects and block as random effect. The statistical model in antioxidant effects included treatment as fixed effect. Data are presented as least-squares means and standard error of the means. Probability values of < 0.05 were considered to be significant.

## 3. Results

### 3.1. Anti-Inflammatory Properties of Mint Oils

**Figure 1:**
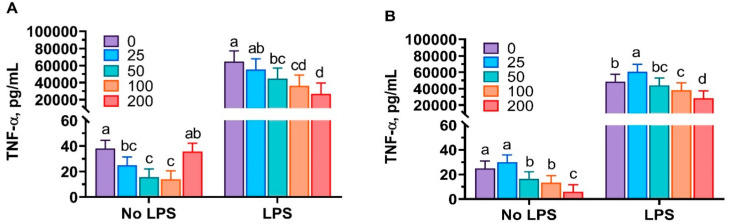
Peppermint oil (**A**) and spearmint oil (**B**) influenced the production of tumor necrosis factor-α (TNF-α; pictogram per milliliter) from porcine alveolar macrophages (PAMs) in the absence or presence of lipopolysaccharide (LPS). Cells were incubated with various concentrations (0, 25, 50, 100, 200 μg/mL) of each mint oil in the presence or absence of LPS (1 or 0 μg/mL) for 24 h. The results were means of values from 6 pigs.

**Figure 2:**
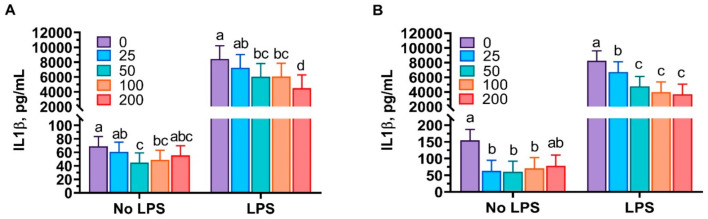
Peppermint oil (**A**) and spearmint oil (**B**) influenced the production of interleukin 1 beta (IL1-β; pictogram per milliliter) from porcine alveolar macrophages (PAMs) in the absence or presence of lipopolysaccharide (LPS). Cells were incubated with various concentrations (0, 25, 50, 100, 200 μg/mL) of each essential oil extract in the presence or absence of LPS (1 or 0 μg/mL) for 24 h. The results were means of values from 6 pigs.

Cell viability was evaluated to identify a range of toxicity of mint oils. In the present study, no cytotoxic effects were observed at the highest dose of both mint oils, because all wells showed ≥ 76% cell viability compared with control cells without LPS challenge (data not shown). The LPS challenge stimulated (*p* < 0.001) the production of TNF-α and IL-1β (Figure 1 and Figure 2). In the absence of LPS, peppermint oil and spearmint oil differently impacted TNF-α production. Administration of peppermint oil quadratically reduced (*p* < 0.05) TNF-α production, while spearmint oil linearly inhibited (*p* < 0.05) TNF-α production from PAMs in the absence of LPS challenge. In the presence of LPS challenge, both peppermint and spearmint oil dose-dependently inhibited (*p* < 0.001) the production of TNF-α (Figure 1).

Both mint oils dose-dependently decreased (*p* < 0.05) the secretion of IL-1β from PAMs in the absence of LPS, while no difference was observed in IL-1β concentration when the highest dose of mint oil was used (Figure 2). The LPS treatment sharply increased (*p* < 0.05) the secretion of IL-1β from PAMs. Treatment with peppermint or spearmint oils significantly inhibited (*p* < 0.05) IL-1β secretion from LPS-challenged PAMs in a dose-dependent manner (Figure 2).

### 3.2. Transepithelial Electrical Resistance of IPEC-J2

**Figure 3:**
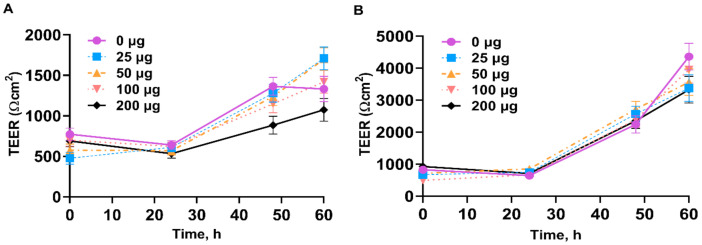
Peppermint oil (**A**) and spearmint oil (**B**) effects on the transepithelial electrical resistance (TEER) of IPEC-J2 cells. The tested doses of peppermint oil (**A**) and spearmint (**B**) were 0, 25, 50, 100, 200 µg/mL.

Cells treated with peppermint and spearmint oils did not exhibit significantly higher transepithelial electrical resistance (TEER) across monolayers at each post-treatment time point compared with controls. IPEC-J2 cells did not exhibit a strong dose response to peppermint and spearmint oils at each time point post-treatment (Figure 3).

### 3.3. Antioxidant Properties of Mint Oils

**Figure 4:**
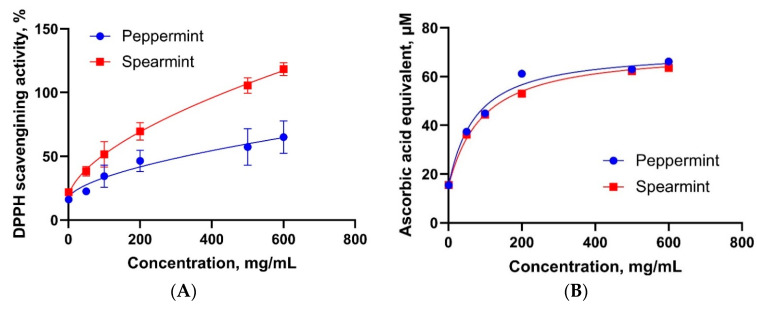
Dose response of peppermint and spearmint oils using chemical-based antioxidant capacity assays. (**A**) 2,2-diphenyl-1-picrylhydrazyl (DPPH) radical scavenging activity assay and (**B**) reducing power assay. Data are presented as the mean of 6 observations.

The antioxidant activity of the two mint oils was determined by assessing their radical scavenging capacity and reducing power. A dose-dependent increase in radical scavenging activity in 2,2-diphenyl-1-picrylhydrazyl (DPPH) assay was observed in spearmint oil and peppermint oil (Figure 4A,B). The strongest response was observed at the highest concentration (600 mg/mL) of each mint oil. However, the maximal plateau was not observed in the DPPH assay for both mint oils. Thus, EC_50_ was not detected in either mint oil (Table 1). Both mint oils displayed increased reducing power in a dose-dependent manner (Figure 4B). The EC_50_ was 71.30 mg/mL for both mint oils. Peppermint oil was less active in the DPPH assay compared to spearmint oil, while the reducing power assay revealed equal results for both mint oils.

### 3.4. Escherichia coli Growth Inhibitory Activity

**Figure 5:**
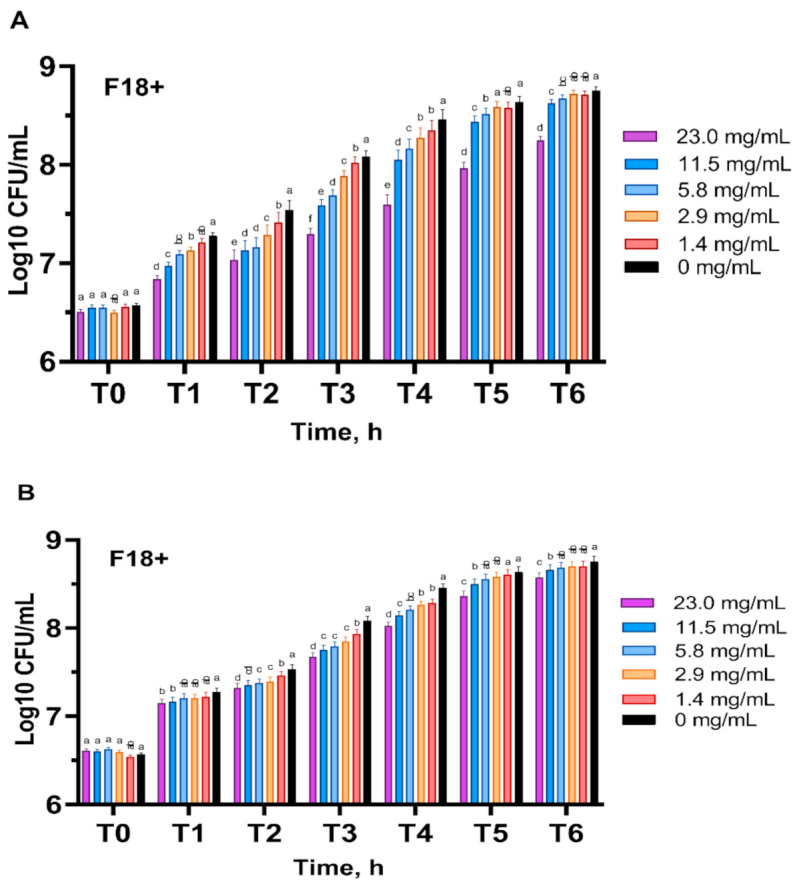
Effects of different concentrations (0, 1.44, 2.87, 5.75, 11.50, and 23.00 mg/mL) of peppermint oil (**A**) and spearmint oil (**B**) on enterotoxigenic *Escherichia coli* F18+ growth in 60 min time intervals (T). Data are expressed as log_10_ CFU/mL LSMEAN ± SEM (*n* = 3). Different superscript letters indicate significant differences at *p* < 0.05 among different concentrations within the same time point.

The growth of the ETEC F18+ strain was tested when treated with different concentrations of peppermint and spearmint oils. The results indicated that ETEC F18+ was sensitive to different doses of mint oils. A dose-dependent effect was observed at each time point for both peppermint and spearmint oil treatments (Figure 5). The highest dose of peppermint oil and spearmint oil (23 mg/mL) showed the maximum inhibitory activity against ETEC F18+ growth at each time point.

## 4. Discussion

The principal objectives of this research were to thoroughly investigate the in vitro biological activity of peppermint and spearmint oils *per se* by measuring their anti-inflammatory properties, antioxidant effects, and inhibitory activity against ETEC F18+. We also aimed to determine whether both mint oils are able to enhance the in vitro intestinal barrier integrity of the IPEC-J2 cell line.

### 4.1. Anti-Inflammatory Properties of Mint Oils

The results of the present study reported that both peppermint and spearmint oils inhibited the production of pro-inflammatory cytokines from LPS-stimulated porcine alveolar macrophages, thus demonstrating the high therapeutic potential of these herbs. Moreover, no cytotoxic effects were observed at the highest doses of both mint oils. The MTT results indicated that the anti-inflammatory effects of peppermint and spearmint oils were not due to the direct killing of cells.

Inflammation is a normal protective response that involves removing dead or damaged host cells when tissue injury or infection occurs. The inflammatory response induces the influx of blood leukocytes, oxidative burst, and release of cytokines [17]. These cytokines, mainly produced by macrophages, are crucial regulators of the host immune response [28]. However, under disease conditions, some cytokines may be overproduced and overexpressed, thereby inducing inflammation and increasing disease symptoms [53,54]. The present study revealed that both peppermint oil and spearmint oil reduced the production and secretion of pro-inflammatory cytokines, such as TNF-α and IL1-β, from macrophages, in line with previous studies [13,55]. Thus, the induction and inhibition of TNF-α and IL1-β produced by mint oils controls the inflammatory response to inflammation against infection and might be beneficial to the host.

Consistent with previously published research, LPS challenge stimulated the production of pro-inflammatory cytokines secreted from macrophages [13,27,28]. The reduction in TNF-α and IL-1β production from LPS-stimulated porcine alveolar macrophages by peppermint and spearmint oil indicated that both mint oils have strong in vitro anti-inflammatory effects. These observations also agree with previously published research, in which the anti-inflammatory effects of other essential oils and plant extracts were reported [13,55,56,57,58,59]. The potential modes of action are proposed to be strongly associated with the inhibition of the nuclear factor kappa-light-chain-enhancer of activated B cells (NF-κB) pathway [60,61,62,63].

### 4.2. Transepithelial Electrical Resistance of IPEC-J2

Transepithelial electrical resistance (TEER) is a strong indicator of the integrity of cellular barriers and tight junction dynamics in epithelial monolayers [33,64]. Various essential oils may improve epithelial barrier function [65,66,67], although the research regarding the effects of mint oils on intestinal barrier function has been limited. In the current study, mint oil treatments did not affect the TEER of the monolayers of IPEC-J2 cells compared with controls. These results indicate that peppermint and spearmint oils may not affect the intestinal epithelial barrier function of IPEC-J2 cells at the tested doses.

### 4.3. Antioxidant Properties of Mint Oils

Appropriate levels of reactive oxygen species (ROS) production are important to maintain redox balance; however, the overproduction of ROS and free radicals triggers oxidative stress, which represents an important chemical mechanism leading to cell damage and cell death [68,69]. Plant extracts can be used as antioxidants in animal feed, and they protect animals from the oxidative stress and cellular damage caused by free radicals [10]. The modes of action of antioxidant compounds include (i) scavenging of free radicals by acting as reducers binding to reactive radicals, (ii) metal chelation, (iii) donating hydrogen atoms or electrons, and (iv) inhibiting prooxidative enzymes [70,71]. Hence, the antioxidant effect of essential oils derived from aromatic plant species such as peppermint and spearmint could retard the formation of free radicals and slow or inhibit the autoxidation process [70,71]. In the current study, both mint oils exhibited consistent antioxidative activity, such as radical scavenging capacity and reducing power activity. These observations were consistent with different studies [72,73,74].

The antioxidant activity of plant extracts relies on their chemical compositions [75]. Mint oils contain phenolic compounds and flavonoids [20,21,22,23,24]. These active compounds act as hydrogen or electron donors to the peroxy radicals, thereby impeding hydroxyl peroxide formation and reducing oxidative damage by scavenging free radicals [76,77]. The reducing power assay estimates the capacity of electron donation by mint oils by measuring their effectiveness in reducing ferric iron to its ferrous form [78]. With increased concentrations, both mint oils displayed enhanced reducing power. These findings are also in agreement with previous works on the *Mentha* genus [79]. Results from the present antioxidant assays are also consistent with another in vitro study, in which different mint oils were evaluated [14].

In brief summary, the current study confirmed the in vitro antioxidant therapeutic activity of peppermint and spearmint oils with two chemical-based assays. In combination with the results of in vitro cellular antioxidant assays [14], we concluded that both mint oils have very promising applications due to their antioxidant activities. However, it is important to consider that the concentration of mint oils used in chemical-based assays may not reflect practical physiological levels when administered in the diet in vivo, and chemical-based assays cannot account for the indirect antioxidant activity in a living organism [14]. Thus, further in vivo study would be needed to justify the antioxidant properties of both mint oils in animal models.

### 4.4. Escherichia coli Growth Inhibitory Activity

*Escherichia coli* growth inhibitory activity results demonstrated that peppermint and spearmint oils inhibited the growth of ETEC F18+ in vitro at different concentrations (0, 1.44, 2.87, 5.75, 11.50, and 23.00 mg/mL) and at different time points (1, 2, 3, 4, 5, 6 h). Higher concentrations were not evaluated because the color of the extracts affects the absorbance reading and might produce false results. The inhibitory effects of peppermint oils were observed very rapidly after treatment, while spearmint oil appeared to exhibit a stable growth inhibitory effect throughout the incubation period. Moreover, both mint oils maintained the inhibitory activity until the end of the analysis (6 h). The highest concentration of both mint oils was the most effective dose against ETEC F18+.

The majority of plant extracts are composed of a high level of phenolic compounds, which are responsible for the wide spectrum of antimicrobial activity against a large variety of pathogenic microorganisms, such as Gram-negative and Gram-positive bacteria [80,81,82,83]. The modes of action responsible for the antimicrobial activity of essential oils are due to their hydrophobic nature. Essential oils significantly bypass the lipids of the bacterial cell membranes, disrupting cell wall structures and increasing the permeability of the bacterial cell membrane [84]. Moreover, active components in essential oils may inhibit the development of virulent structures in bacteria [85] or may interrupt the enzyme functions of bacteria associated with their virulence [86]. The antimicrobial mechanism of action varies with the type of essential oils and their composition, the concentration of active substances, and the strain of the tested microorganism [87].

The high growth inhibition of mint oils against different bacterial pathogens has been confirmed in numerous studies. The literature has reported their growth inhibition and antimicrobial activity against *Micrococcus luteus*, *Salmonella typhimurium* [88], *Staphylococcus aureus* [89], *Escherichia coli* [34,89,90,91], and *Pseudomonas aeruginosa* [91]. Moreover, the study of Muntean et al. [38] highlighted the presence of bactericidal activity on an extensively drug-resistant strain of *Escherichia coli*. These cases in the literature were in line with our results.

Although the data from the current study need further support from additional studies to evaluate the synergistic effect of both mint oils, the results from the present study are very encouraging and indicate that these herbs should be studied more extensively in the pig industry due to their potential as therapeutic antibacterial agents.

## 5. Conclusions

A ban on the use of antibiotics as growth-promoting agents in swine production was implemented in the European Union in 2006. Thus, novel additives such as plant extracts and phytochemicals are of interest to replace in-feed antibiotics. The results of the current study emphasize the in vitro anti-inflammatory, antioxidant, and anti-microbial activities of two mint oils extracted from peppermint and spearmint. Most importantly, the strong inhibitory effects of both mint oils on ETEC F18+, one of the most common pathogens responsible for post-weaning diarrhea and increased antibiotic treatment, demonstrate that these mint oils are promising candidates to replace antibiotics in feed. Although peppermint and spearmint oils did not affect TEER in IPEC-J2 cells in vitro, this does not exclude their potential impacts on the gut integrity of pigs if overall gut health can be improved in vivo. More research is necessary to further explore the therapeutic potential and future perspectives of essential oils extracted from mint and other herbs to deal with multifactorial diseases in the pig industry.

## Figures and Tables

**Table 1:** The half-maximal effective concentration of peppermint and spearmint oils measured by chemical-based antioxidant activity assays.

Assay ^2^	EC_50_^1^ (Goodness of Fit), mg/mL	
	Peppermint Oil	Spearmint Oil
DPPH scavenging activity	Not detected	Not detected
Reducing power assay	71.30 (0.7765)	71.30 (0.9722)

^1^EC_50_ = half maximal effective concentration. ^2^ DPPH = 2,2-diphenyl-1-picrylhydrazyl.

## Data Availability

Data are contained within the article.
